# Supplementary figures and images for: Using species distribution models to locate the potential cradles of the allopolyploid Gypsophila bermejoi G. López (Caryophyllaceae)
Source: PLoS One. 2020 May 19;15(5):e0232736. doi: 10.1371/journal.pone.0232736 (PMC7237017; doi:10.1371/journal.pone.0232736)

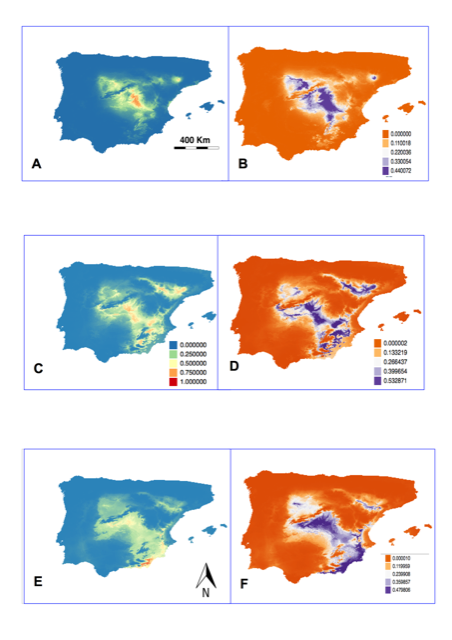

Supplement: S1 Fig — (TIFF) [file pone.0232736.s001.tiff]

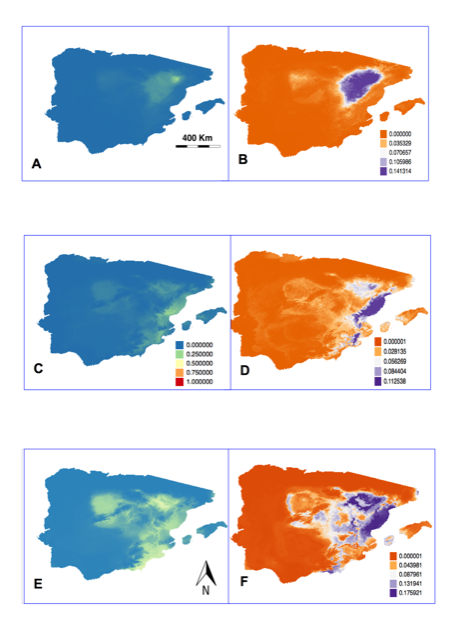

Supplement: S2 Fig — (TIFF) [file pone.0232736.s002.tiff]

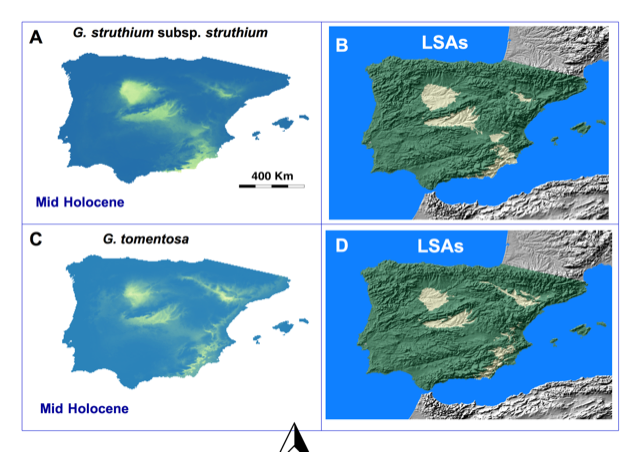

Supplement: S3 Fig — For this climatic period, the MaxEnt models for G. struthium subsp. struthium and G. tomentosa are shown in A and C respectively. current distribution of this plant according to our hypothesis. B and D show the LSAs for both taxa during the Mid Holocene. (TIFF) [file pone.0232736.s003.tiff]
