# Supplementary material for: Using species distribution models to locate the potential cradles of the allopolyploid Gypsophila bermejoi G. López (Caryophyllaceae)
Source: PLoS One. 2020 May 19;15(5):e0232736. doi: 10.1371/journal.pone.0232736 (PMC7237017; doi:10.1371/journal.pone.0232736)
Supplement: Attachment — Submitted filename: PONE-D-19-14999_reviewer.pdf [file pone.0232736.s004.pdf]

PLOS ONE

Using Species Distribution Models to locate the potential cradles of the allopolyploid Gypsophila bermejoi G. López (Caryophyllaceae)

--Manuscript Draft--

**Manuscript Number:**	PONE-D-19-14999
**Article Type:**	Research Article
**Full Title:**	Using Species Distribution Models to locate the potential cradles of the allopolyploid Gypsophila bermejoi G. López (Caryophyllaceae)
**Short Title:**	An approach to locate the potential cradles of the allopolyploid Gypsophila bermejoi
**Corresponding Author:**	Carmen Bartolomé, Ph.D.<br>Universidad de Alcala de Henares<br>Alcala de Henares, Madrid SPAIN
**Keywords:**	
**Abstract:**	Polyploidy has been an influential force in plant evolution, playing a crucial role in diversification. Differences in polyploid and diploid distributions have been long noted, with polyploid taxa especially abundant in harsh environments. These plants have higher photosynthetic rates and/or higher tolerance to water deficits. Moreover, there is data pointing to an increase in the rate of unreduced gamete formation by plants under conditions of stress. Accordingly, a higher frequency of polyploid individuals would be expected in populations living under extreme environments, a phenomenon that may be relevant when considering the origin of allopolyloid species. Hybridization between distinct autopolyploids is known to produce allopolyploids and hence, a high frequency of compatible autopolyploids in an area could enhance the formation of stable populations of the corresponding allopolyploid hybrid. Here we consider the allopolyploid species Gypsophila bermejoi G. López and its parental taxa G. struthium L. subsp. struthium and G. tomentosa L. We have used MaxEnt models to locate areas with low bioclimatic suitability for both parental taxa during the Last Glacial Maximum (LGM), hypothesizing that the rate of tetraploid hybrid formation would be higher than expected where low suitability areas of both parental species overlap. We selected those areas taking into account the strict gypsophyllic nature of these taxa. There is data pointing to a post-glacial origin of the current G. bermejoi populations and according to our hypothesis, such locations could be centers for hybrid tetraploid formation or potential cradles of this species. Indeed, potential Mid-Holocene cradles were also identified in this manner. The evolution of bioclimatic suitability in both LGM and Mid-Holocene cradles was studied to assess the possible survival of the hybrids, and the current distribution of G. bermejoi proved to be consistent with our hypothesis.
**Order of Authors:**	Miguel de Luis
	Julio Álvarez-Jiménez
	Francisco Javier Rejos-Ballesteros
	Carmen Bartolomé, Ph.D.
**Additional Information:**	
**Question**	**Response**
**Financial Disclosure**<br><br>Enter a financial disclosure statement that describes the sources of funding for the work included in this submission. Review the submission guidelines for detailed requirements. View published research articles from *PLOS ONE* for specific examples.	NO

Powered by Editorial Manager® and ProduXion Manager® from Aries Systems Corporation

This statement is required for submission and **will appear in the published article** if the submission is accepted. Please make sure it is accurate.<br><br>**Unfunded studies**<br>Enter: *The author(s) received no specific funding for this work.*<br><br>**Funded studies**<br>Enter a statement with the following details:<br>• Initials of the authors who received each award<br>• Grant numbers awarded to each author<br>• The full name of each funder<br>• URL of each funder website<br>• Did the sponsors or funders play any role in the study design, data collection and analysis, decision to publish, or preparation of the manuscript?<br>• **NO** - Include this sentence at the end of your statement: *The funders had no role in study design, data collection and analysis, decision to publish, or preparation of the manuscript.*<br>• **YES** - Specify the role(s) played.<br><br>* typeset	
**Competing Interests**<br><br>Use the instructions below to enter a competing interest statement for this submission. On behalf of all authors, disclose any competing interests that could be perceived to bias this work—acknowledging all financial support and any other relevant financial or non-financial competing interests.<br><br>This statement **will appear in the published article** if the submission is accepted. Please make sure it is accurate. View published research articles from *PLOS ONE* for specific examples.	NO authors have competing interests

Powered by Editorial Manager® and ProduXion Manager® from Aries Systems Corporation

**NO authors have competing interests**<br><br>Enter: *The authors have declared that no competing interests exist.*<br><br>**Authors with competing interests**<br><br>Enter competing interest details beginning with this statement:<br><br>*I have read the journal's policy and the authors of this manuscript have the following competing interests: [insert competing interests here]*<br><br>* typeset	
**Ethics Statement**<br><br>Enter an ethics statement for this submission. This statement is required if the study involved:<br><br>• Human participants<br>• Human specimens or tissue<br>• Vertebrate animals or cephalopods<br>• Vertebrate embryos or tissues<br>• Field research<br><br>Write "N/A" if the submission does not require an ethics statement.<br><br>General guidance is provided below. Consult the submission guidelines for detailed instructions. **Make sure that all information entered here is included in the Methods section of the manuscript.**	N/A

Powered by Editorial Manager® and ProduXion Manager® from Aries Systems Corporation

**Format for specific study types**<br><br>**Human Subject Research (involving human participants and/or tissue)**<br>• Give the name of the institutional review board or ethics committee that approved the study<br>• Include the approval number and/or a statement indicating approval of this research<br>• Indicate the form of consent obtained (written/oral) or the reason that consent was not obtained (e.g. the data were analyzed anonymously)<br><br>**Animal Research (involving vertebrate animals, embryos or tissues)**<br>• Provide the name of the Institutional Animal Care and Use Committee (IACUC) or other relevant ethics board that reviewed the study protocol, and indicate whether they approved this research or granted a formal waiver of ethical approval<br>• Include an approval number if one was obtained<br>• If the study involved *non-human primates*, add *additional details* about animal welfare and steps taken to ameliorate suffering<br>• If anesthesia, euthanasia, or any kind of animal sacrifice is part of the study, include briefly which substances and/or methods were applied<br><br>**Field Research**<br><br>Include the following details if this study involves the collection of plant, animal, or other materials from a natural setting:<br>• Field permit number<br>• Name of the institution or relevant body that granted permission	
**Data Availability**<br><br>Authors are required to make all data underlying the findings described fully available, without restriction, and from the time of publication. PLOS allows rare exceptions to address legal and ethical concerns. See the PLOS Data Policy and FAQ for detailed information.	Yes - all data are fully available without restriction

A Data Availability Statement describing where the data can be found is required at submission. Your answers to this question constitute the Data Availability Statement and **will be published in the article**, if accepted.<br><br>**Important:** Stating 'data available on request from the author' is not sufficient. If your data are only available upon request, select 'No' for the first question and explain your exceptional situation in the text box.<br><br>Do the authors confirm that all data underlying the findings described in their manuscript are fully available without restriction?	
**Describe where the data may be found in full sentences. If you are copying our sample text, replace any instances of XXX with the appropriate details.**<br><br>• If the data are **held or will be held in a public repository**, include URLs, accession numbers or DOIs. If this information will only be available after acceptance, indicate this by ticking the box below. For example: *All XXX files are available from the XXX database (accession number(s) XXX, XXX.*).<br>• If the data are all contained **within the manuscript and/or Supporting Information files**, enter the following: *All relevant data are within the manuscript and its Supporting Information files.*<br>• If neither of these applies but you are able to provide **details of access elsewhere**, with or without limitations, please do so. For example:<br><br>*Data cannot be shared publicly because of [XXX]. Data are available from the XXX Institutional Data Access / Ethics Committee (contact via XXX) for researchers who meet the criteria for access to confidential data.*<br><br>*The data underlying the results presented in the study are available from (include the name of the third party*	All relevant data are within the manuscript and its Supporting Information files.

and contact information or URL).<br>• This text is appropriate if the data are owned by a third party and authors do not have permission to share the data.<br><br>* typeset	
Additional data availability information:	

Manuscript Click here to download Manuscript Potential_Cradles_G4.docx

Using Species Distribution Models to locate the potential cradles of the allopolyploid *Gypsophila bermejoi* G. López *(Caryophyllaceae)*

Miguel de Luis[1], Julio Álvarez-Jiménez[1], Francisco Javier Rejos-Ballesteros[1], Carmen Bartolomé[1]*

1. Departamento de Ciencias de la Vida. Facultad de Ciencias. Universidad de Alcalá. Alcalá de Henares (Madrid). Spain.

* Corresponding autor
E-mail: carmen.bartolome@uah.es

Using Species Distribution Models to locate the potential cradles of the allopolyploid *Gypsophila bermejoi* G. López *(Caryophyllaceae)*

ABSTRACT

Polyploidy has been an influential force in plant evolution, playing a crucial role in diversification. Differences in polyploid and diploid distributions have been long noted, with polyploid taxa especially abundant in harsh environments. These plants have higher photosynthetic rates and/or higher tolerance to water deficits. Moreover, there is data pointing to an increase in the rate of unreduced gamete formation by plants under conditions of stress. Accordingly, a higher frequency of polyploid individuals would be expected in populations living under extreme environments, a phenomenon that may be relevant when considering the origin of allopolyloid species. Hybridization between distinct autopolyploids is known to produce allopolyploids and hence, a high frequency of compatible autopolyploids in an area could enhance the formation of stable populations of the corresponding allopolyploid hybrid. Here we consider the allopolyploid species *Gypsophila bermejoi* G. López and its parental taxa *G. struthium* L. subsp. *struthium* and *G. tomentosa* L. We have used MaxEnt models to locate areas with low bioclimatic suitability for both parental taxa during the Last Glacial Maximum (LGM), hypothesizing that the rate of tetraploid hybrid formation would be higher than expected where low suitability areas of both parental species overlap. We selected those areas taking into account the strict gypsophyllic nature of these taxa. There is data pointing to a post-glacial origin of the current *G. bermejoi* populations and according to our hypothesis, such locations could be centers for hybrid tetraploid formation or potential cradles of this species. Indeed, potential Mid-Holocene cradles were also identified in this manner. The evolution of bioclimatic suitability in both LGM and Mid-Holocene cradles was studied to assess the possible survival of the hybrids, and the current distribution of *G. bermejoi* proved to be consistent with our hypothesis.

INTRODUCTION

Polyploidy or Whole Genome Duplication (WGD) is an ubiquitous and influential force in plant evolution, both on macro- and micro-evolutionary timescales. [**1, 2**] WGD could have played a crucial role in angiosperm evolution and in the diversification of their main lineages. Indeed, some ancient WGD events are associated with high rates of diversification, a remarkable example of which is related to the origin of *Asteraceae*, a family of angiosperms containing one of the largest number of species [**3**,**4,5,6**].

Polyploidy can arise in two ways. Allopolyploidy takes place when the genomes are derived from

different species, individuals that can be produced by interspecific hybridization. By contrast, autopolyploidy is the result of intraspecific WGD events and it has long been considered to be less common, although it is true that autopolyploids are more difficult to detect. Moreover, if the rate of tetraploid formation were comparable to the mutation rate, many more allopolyploids would be expected in nature [**7**,**8**].

Differences in the distribution of polyploids and diploids have long been noted [**9**]. Polyploid taxa are frequently found in plant communities worldwide and they seem especially abundant in challenging environments, such as at high latitudes, at high elevations or in arctic areas [**9**,**14**]. Such distributions are consistent with observations regarding the physiological changes in polyploid plants, and when changes in body size follow genome duplication, it is common to find that "morphological differences *could result in different adaptations of cytotypes*" (emphasis ours) [**15**,**16**]. Other central processes in plant physiology may also be affected by genome duplication and there is evidence that polyploids display a higher tolerance to water deficit [**17**] or higher photosynthetic rates [**18**]. One recurrent observation in natural polyploid plants is that they usually show improved growth and better adaptation to harsh environments, consequently displaying evolutionary advantages [**19**]. These observations explain the higher frequency of polyploids proposed in extreme environments [**20**].

It is known that the rate of unreduced gamete formation increases under conditions of stress in plants [**21**, **22**], such as extreme cold, highly fluctuating temperatures, nutritional and water stress, amongst others [**21**]. In areas where such challenging conditions prevail, small populations would be expected and this circumstance could further enhance the establishment of polyploids [**23**]. Interestingly, polyploids often have a higher rate of self-fertilization, a higher capacity to propagate clonally and a perennial life-cycle. All these traits would improve their chances of establishing stable populations in areas where conditions are non-optimal [**20**,**24**,**25**]. The higher frequency of polyploid individuals in non-optimal areas or extreme bioclimatic conditions, if true, could be relevant to the origin of allopolyploid species. Moreover, it is known that hybridization between distinct autopolyploids directly produces allopolyploids [**21**] and according to this observation, the presence of a high frequency of compatible autopolyploids in an area could trigger the formation of stable populations of the corresponding allopolyploid hybrid.

In terms of species distribution models (SDMs), all the above observations could mean that the frequency of polyploid individuals increases at the periphery of species ranges [**26-29**], where the suitability for the species is low but not zero. Such Low Suitability Areas (LSAs) would harbor populations with a higher frequency of autopolyploids. If we consider two interbreeding species, we would expect a higher formation of allopolyploids in areas where the periphery of both ranges overlap. If there is a higher frequency of autopolyploids, the probability of producing non-reduced gametes would also be higher [**22**], increasing the probability of fertile interspecific hybrids arising. Thus, we could consider these areas as more favorable sites to establish stable allopolyploid populations (Fig. 1), and such locations could act as potential formation centers or cradles for the hybrid species.

G. bermejoi is an allopolyploid species derived from the parental *G. struthium* L. subsp*. struthium* and *G. tomentosa* L [**30**]. All these plants are gypsophytes endemic to the Iberian Peninsula, and they are of particular ecological, evolutionary and biochemical interest. Some evidence points to a recent origin of the current populations of this taxon [**31,32**]. Indeed, based on Species Distribution Models (SDMs) and niche modelling it has been shown that the parental taxa of *G. bermejoi* had a high degree of sympatry during the Last Glacial Maximum (LGM), although the climatic conditions repressed the formation of this species because of its very narrow bioclimatic niche at that time [**31,32**]. We have used SDMs to locate the LSAs of the parental species of *G. bermejoi* and then, using a Geographical Information System (GIS),we have found the areas where these LSAs overlap. According to our hypothesis those areas could be potential cradles for *G. bermejoi* speciation (Fig. 1). SDMs are useful tools to visualize the ecological preferences of a species within a specific area and they have been used successfully to examine a wide range of ecological issues. In a number of studies they have also been used to evaluate the effects of climate change on ecosystems and communities, or the effectiveness of conservation plans [**33,40**].

Figure 1. A possible mechanism of cradle formation. We show two closely related species with an allopatric distribution. Both of them have a high suitability area around the centroid of their ranges (regions *A* and *B* in 1) and a lower suitability area (LSA) around their ranges (shadowed areas *a* and *b* in 1). According to a number of observations (see text), a higher frequency of autopolyploids (solid squares and circles) could be expected in the peripheral regions of both ranges (the LSAs). The LSAs will overlap if both species have some degree of sympatry and hence, there would be regions with a stronger presence of autopolyploids emanating from both species. This circumstance would favor the formation of fertile tetraploid hybrids (red triangles, species C in 3). We considered such areas as potential cradles or potential formation centers for the hybrid species. Such hybrids could also arise at other locations where the parental taxa could interbreed, although a lower rate of hybrid formation would be expected. This is a possible scenario for the speciation of *G. bermejoi*, where the A and B species would be its parental *G. struthium* subsp. *struthium* and *G. tomentosa*.

The idea of a stronger presence of polyploids in harsh environments is not new, yet there is still much to learn about this phenomenon. Here we use this feature as the basis to establish our hypothesis that the potential cradles of the hybrid species *Gypsophila bermejoi* G. López [**30**] are gypsum outcrops in areas where the LSAs of its parental taxa overlap. We located a set possible centers where the climatic conditions during the (LGM) and the mid-Holocene (MH) period would favor the formation of this species, and we assessed the evolution of the bioclimatic conditions at those locations to determine which populations species could have survived. Finally, we compared these results with the current locations of *G. bermejoi*. Our main goal was to find out if this working

hypothesis could to some extent predict the current distribution of the species.

Materials and Methods

Study area and species

All the species included in this work are endemic to the Iberian Peninsula, which is located in the southwestern part of Europe, between latitudes 36°00'08"N - 43°47'38"N and longitudes 9°29'00"O - 3°19'00"E. With an area of approximately 597,000 km^2, this is the second largest peninsula on the continent, and it includes most of the territory of Spain and Portugal. In this study, we focused on the gypsum steppes within the Peninsula, containing the gypsum outcrops in this territory and covering a total extension of 32,487 km^2 (5.4% of the Peninsula) [**41**]. These habitats are concentrated in the eastern half of the Peninsula, under the influence of a Mediterranean climate.

Our research focuses on the speciation of *Gypsophila bermejoi,* a natural hybrid derived from *G. struthium* subsp*. struthium* (2n=34) and *G. tomentosa* (2n=34) [**30, 44**]. This genus is included in the *Caryophyllaceae* family of plants, and it is an allopolyploid species (2n=68) found on verges and slopes in areas of gypsum soil [**41**]. While the range of *G. bermejoi* overlaps that of its parental taxa to some degree and all these plants are gypsophytes endemic to the Iberian Peninsula [**42**], the ecological niche of *G. bermejoi* shows noticeable differences [**32**]. Gypsum soils are harsh environments for angiosperms due to their extreme xericity, severe nutritional imbalances and the presence of a crusty surface [**42,43**]. In biochemical terms, gypsophytes can produce a wide range of secondary metabolites and they have a tendency to accumulate certain minerals. The production of saponins and antioxidants, like that of phenols and flavonoids, has been studied widely in *Gypsophila* [**44–46**]. As a result of the particular ecological conditions of the gypsisols, the peninsular gypsum outcrops harbor plants that are highly adapted to their environment, and endemic species are common at these sites. This circumstance and the patchy nature of these habitats makes these species a good model to study plant evolution and speciation processes [**43**].

Data Sources

Maximum Entropy (MaxEnt) modeling of ecological niches requires two types of data for the study area: species occurrence and environmental data. Here, we used species occurrence data from the Global Biodiversity Information Facility (GBIF) database [**47**] and a set of WorldClim bioclimatic variables. WorldClim is a free database with sets of global climatic data that can be used for ecological modeling and mapping (gridded climate data), with a spatial resolution of about 1 km^2. The internet portal (www.worldclim.org) offers publically data for 19 bioclimatic variables [**48**] ~~and the data are available as a set of global climatic layers that can be used as raster files in a GIS~~. ~~Past and future projections were generated using different climate models and these data can be~~

~~downloaded for free.~~ We also used bioclimatic maps for the MH period and LGM generated through the Community Climate System Model (CCSM4). CCSM4 combines four separate models of the atmosphere, land, oceans and sea-ice, generating a simulation of the global climate [**49**].

Modeling species distributions using the MaxEnt approach requires correlations between the predictors to be minimized [**50**]. From a set of available predictors, we selected those with the lowest Pearson correlation coefficient using a clustering algorithm to produce a dendrogram that groups the variables according to their degree of correlation. A jack-knife test of the contribution of the variables to the model was also performed. After combining the information obtained through correlation dendrogram and jackknife for variable contribution, and taking into account the abiotic factors determining the vegetation patterns in a semi-arid Mediterranean landscape [**51,52**], we selected from the less correlated variables those with the highest contribution, and one related with precipitation values during the dry season [**60**]

Table 1. List of the environmental variables selected to implement both the MaxEnt species distribution models.

Variable	Source	Resolution
bio4 - the dispersion of seasonal temperatures	WorldClim	2.5’
bio6 – the minimum temperature in the coldest month	WorldClim	2.5’
bio13 – the rainfall in the wettest month	WorldClim	2.5’
bio14 – the rainfall in the driest month	WorldClim	2.5’

Maximum Entropy (MaxEnt) Model

To model ecological niches and potential distributions we used the MaxEnt approach [**53**,**54**], the algorithms for which are currently implemented in R language [**55**] and using a graphic interface (available at: http://www.cs.princeton.edu/~schapire/maxent/). The most recent version of the program available was used here (MaxEnt 3.4.1: for an explication of the method see [**56,57**]). This tool essentially assesses the probability of the presence of a species in function of the environmental variables selected. The inputs are data files on the presence of the species and a series of maps of the environmental variables considered to be significant. The results can be interpreted as an index of suitability for the particular species to that habitat [**54**]. We dealt with autocorrelation issues by eliminating any redundant presence in each pixel on the scale of the bioclimatic variables used. We used 70% of the presence records to train the models and 30% to test them. The study focused on the effect of climatic oscillations and as such, we used only bioclimatic variables to generate the models. Given the strict gypsophyllic nature of these plants [**58**], they are only present on gypsum

soils and their suitability on other soils is “0”. Therefore, we applied the MaxEnt model with climate variables to the entire Iberian Peninsula and we then subtracted the gypsum soil territories to evaluate the suitability areas. In choosing the functions “feature”, we deactivated the functions “hinge” and “threshold”, thereby obtaining curves that were easier to interpret and that were better adjusted to the ecological theory of the niches [**57**]. We validated the MaxEnt models by means of the Area Under the Curve (AUC) and the True Skill Statistic (TSS) [**57**-**59**].

An approach to find potential *G. bermejoi* cradles

As pointed out previously, a number of observations seem to support the hypothesis of a higher frequency of polyploid individuals in the peripheral domains of the ranges for a given species [**26-29**], where the suitability for the species is weaker. If this statement is true, higher autopolyploid frequencies of would be expected at the periphery of the potential areas predicted by the MaxEnt models. Here we used this idea to predict possible centers of *G. bermejoi* formation. As indicated, our hypothesis is that the gypsum outcrops where the LSAs of both parental taxa overlap (suitable soils with higher frequencies of *G. struthium* subsp. *struthium* and *G. tomentosa* autopolyploids) would be potential cradles for the allopolyploid species *G. bermejoi*. As such, we assessed whether the results obtained were consistent with the current distribution of *G. bermejoi*.

Our approach to find potential cradles is summarized as:

1- Generating Species Distribution Models (SDM). The MaxEnt approach was used to produce suitability models based on a series of bioclimatic variables carefully selected (see above). These models were then projected onto the geographical space of the Iberian Peninsula in the current climatic conditions, and in two past climatic ages: the LGM and MH period. We generated these models for *G. bermejoi* and for its parental taxa, *G. struthium* subsp*. struthium* and *G. tomentosa*.

2- Obtaining a statistical distribution of suitability values. To achieve our goal it is crucial to define what is “low” suitability. To do so, we carried out an exploratory analysis of the suitability values predicted by the models under the current climatic conditions for both parental taxa. A statistical distribution of these bioclimatic suitability values was obtained, considering only the sites where they are currently present. In other words, we took the readings of the suitability values predicted by the models at the locations where the species is currently found. Our goal here was to define the lowest suitability values predicted by the model at the sites where both taxa occur (*G. struthium subsp. struthium* and *G. tomentosa*). It is important to bear in mind that by doing so, we obtain the lowest suitability values predicted by the SDM for the parental taxa at locations where those plants really grow (Figure 2A).

Figure 2. Low Suitability Areas (LSA) for *G. struthium* subsp. *struthium* and *G. tomentosa*. To establish the intervals of low suitability, we need to know the suitability values predicted by the models at the locations where these taxa are present under current climatic conditions.

For each taxon, we considered low suitability values to be all those between the minimum and the first quartile. We tried to assess the suitability values in which a high frequency of autopolyploids is expected for both species. In Figure 2A, the suitability value histograms for both taxa are shown, with the minimum and first quartiles indicated by colored lines. In interval 1 (the minimum between *G. tomentosa* and *G. struthium* subsp. *struthium*), there is low suitability for *G. tomentosa* but no presence of *G. struthium* subsp. *struthium* (making interbreeding impossible). In interval 2 (between the minimum and the first quartile of G. struthium subsp. struthium) there is low suitability for both taxa and according to our hypothesis, a higher frequency of autoplyploids might be expected. Interval number 3 has low suitability for *G. tomentosa* but not for *G. struthium* subsp. *struthium*. If we accept the situations for low suitability, there could be a higher frequency of *G. tomentosa* autopolyploids in this interval but not of *G. struthium* subsp. *struthium*. In these conditions a higher frequency of triploid individuals would be expected, which could also enhance the formation of the *G. bermejoi* tetraploid hybrid. As a consequence, we considered the 2+3 interval to be favorable for the formation of *G. bermejoi*, between the higher minimum (that of *G. struthium* subsp. *struthium*) and the higher first quartile (that of *G. tomentosa*). Figures 2B and 2D show the MaxEnt models during the LGM for *G. struthium* subsp. *struthium* and *G. tomentosa*, respectively, whilst figures 2C and 2E show the LSAs for both taxa (suitability values between 0.1188 and 0.46744).

3- Defining Low suitability intervals. At this point we need to establish a threshold to define what can be considered “low”. To do so, we used the range between the minimum and the first quartile of the statistical distributions for the suitability values of the parental taxa (**Table 2**). We considered a low suitability interval to be that defined between the minimum and the first quartile of the suitability values predicted by the SDM at the current locations of both taxa (*G. tomentosa* minimum = 0.0356, first quartile = 0.46744 and *struthium struthium* minimum = 0.1188, first quartile = 0.4372). It is important to bear in mind that our aim is to assess the suitability interval that could enhance the formation of allopolyploids. In other words, the suitability values favoring a higher frequency of autopolyploids for at least one of the parental taxa. We used the same interval for both taxa and we established the interval between the lowest minimum and the highest first quartile (Figure 2A). We have to consider this step by step, whereby below the lowest minimum no taxon is present and obviously, we must discard that interval. Between both minima, we had low suitability for one taxon and no presence for the other, making interbreeding impossible. The interval defined by the *G. struthium* subsp. *struthium* minimum and its first quartile represents a low suitability for both parental taxa (Figure 2A). In these conditions higher frequencies of autopolyploids could be expected for both taxa, as well as a higher rate of allopolyploid formation. However, we also considered the interval between the first quartiles of *G. struthium* subsp. *struthium* (0.4372) and *G. tomentosa* (0.46744). If the working hypothesis were correct, there would be a higher frequency of autopolyploids in this

interval for just one taxon. However, according to the triploid bridge mechanism **[60, 61]**, this interval could also facilitate the formation of allopolyploids. As a consequence, we consider that the bioclimatic suitability interval between the first quartile of *G. struthium* subsp. *struthium* minimum (0.4372) and *G. tomentosa* (0.46744) could favor the formation of *G. bermejoi* allopolyploid species. Such intervals correspond to a series of pixels in the maps defining the LSAs for each taxon. The next logical step is to locate those regions in the geographical space and to find where the LSAs for the parental taxa overlap.

4- Low Suitability Areas in the geographic space for the LGM. Once the low suitability interval is defined, a GIS can be used to establish the LSAs for the parental species of *G. bermejoi* during the LGM, for which we used QGIS [**62**]. From the potential area predicted for *G. struthium* subsp. *struthium* and *G. tomentosa* during the LGM (Figures 2B and 2D), we selected those areas with suitability values between 0.1188 and 0.46744 (Figures 2C and 2E). According to our working hypothesis, those areas should have more autopolyploid plants and if so, a higher frequency of allopolyploids would be expected in the locations where both these areas overlap (Figure 3A). A stronger presence of non-reduced gametes in such populations would facilitate the formation of the fertile hybrid *G. bermejoi*. Thus, we used QGIS to establish where the LSAs for the parental taxa overlap.

5- Filtering for locations with suitable soils: locating the LGM cradles. All the species considered in this work are strict gypsophytes. So far we have located the areas where bioclimatic suitability could favor the formation of *G. bermejoi* but we have yet to take into account the special edaphic conditions required by this plant. To select the sites with suitable soils, we used the current distribution of *Ononis tridentata* (Figure 3A), a common gypsum soil indicator species with a broad niche [**63-64**]. Using QGIS and an algorithm implemented in R programming language [**55**], we filtered the sites where *Ononis tridentata* was present to identify areas where the low bioclimatic suitability of the parental taxa overlap (Figures 3B and 3C). We consider these locations as potential cradles for *G. bermejoi* and in such places, the presence of two concomitant factors could favor the establishment of stable populations of this species: suitable soils and according to the working hypothesis, a high frequency of polyploid individuals from the parental *G. bermejoi* taxa.

Figure 3. The presence of the common gypsum indicator *O. tridentata* was used to plot the sites of gypsum soils on the map *(A)*. During the LGM, the LSAs of the *G. bermejoi* parental taxa overlapped at some locations due to the high degree of sympatry of both taxa (lime colored areas in *B* and *C*). According to our hypothesis, higher frequencies of autopolyploids and non-reduced gametes would be expected in those areas. The three taxa studied are strict gypsophytes and hence, we filtered all the gypsum outcrops included in the areas where the LSAs for both parental taxa (*G. struthium* subsp. *struthium* and *G. tomentosa*) overlap (*C*). At those sites, there would be a higher probability of establishing thriving populations of the fertile *G. bermejoi* hybrid and thus, we consider such places as potential cradles for this

species. (*D*) The overlapping LSAs of the parental *G. bermejoi* taxa during the MH period and (*E*) the potential cradles for the same climatic period.

6- Locating potential cradles for the Mid-Holocene period. The SDM models show dramatic changes in the potential distribution for the species studied during the MH period. New potential bioclimatic areas mean new LSAs and hence, new potential cradles. To locate these potential cradles, we repeated the protocol using the MaxEnt models for the MH period (Figure 3D, 3E): the LSAs for the MH period can be seen in the supplementary materials section (S1).

Predicting the expected distribution of *G. bermejoi* under current climatic conditions

Having determined the potential *G. bermejoi* cradles, it is possible to forecast its expected current distribution by studying the evolution of the bioclimatic suitability of this taxon at such locations, or in other words, by foreseeing the fate of such potential cradles. Our goal here is to find out which of these locations could have maintained stable populations of *G. bermejoi* despite the dramatic climatic changes that have occurred in the past 25,000 years. By doing so, we could assess which potential cradles might have survived such climatic oscillations and could currently harbor *G. bermejoi* populations. Hence, we assessed in which of these cradles bioclimatic suitability remained reasonably high for *G. bermejoi* as follows:

1- Defining a survival threshold for *G. bermejoi*. A “survival” threshold was defined in order to establish which of the possible cradles could have maintained *G. bermejoi* populations up to the present day. To do so, we generated a MaxEnt model for *G. bermejoi* under current climatic conditions and we took the suitability values at the points of occurrence of this species. The resulting distribution is shown as a histogram in figures 4A to 4D (lower histograms) and the minimum suitability value of this distribution was used as a “survival” threshold.

Figure 4. The bioclimatic suitability of *G. bermejoi* cradle locations in the LGM and Mid-Holocene periods were evaluated in the Mid-Holocene and current climate conditions. The resulting statistical distributions were plotted as the dark blue histograms, while the pale blue histogram corresponds to the suitability values of the MaxEnt model at the sites the plant occurs under current climate conditions. The minimum value of this distribution was used as a survival threshold for this species. Comparing both histograms, we can see how many potential cradles maintain a bioclimatic suitability below this minimum and thus, *G. bermejoi* could not endure the climatic conditions at these sites. “*A*” shows the comparison between *G. bermejoi* suitability values and those of the LGM cradles under Mid-Holocene climatic conditions and “*B*” those for the current climatic conditions. “*C*” compares the *G.*

***bermejoi* suitability values with those of the Mid-Holocene cradles during the Mid-Holocene and *"D"* under current climatic conditions. In all cases, the vast majority of cradles produced very low suitability values for *G. bermejoi* and it is difficult to consider the survival of this species at such locations.**

2- Evolution of the bioclimatic suitability conditions for *G. bermejoi* at the cradles. The evolution of bioclimatic suitability of the potential cradles as a whole was studied from the LGM to the present (Figure 4). We treated the potential cradles generated during the LGM (Figures 4A and 4B) separately from those generated in the Mid-Holocene (Figures 4C and 4D). The former were studied at three moments: LGM, MH period and current climatic conditions. Obviously, we only studied suitability during the MH and the current climate for the MH cradles. We then took the suitability values of *G. bermejoi* produced by MaxEnt models at the LGM cradles for the MH and current climatic conditions. Comparative histograms were generated to get an idea of the potential cradles where the suitability values for *G. bermejoi* remained below the minimum for different climatic periods (Figure 4), sites at which *G. bermejoi* populations would be doomed. This approach also enabled us to filter the surviving cradles at different moments in the past and under the present climatic conditions.

3- Filtering the potential cradles in the geographical space: an expected current distribution prediction. Two series of maps were generated using QGIS [**62**] to depict the fate of both the LGM and MH potential cradles (Figures 5B to 5D and 5F to 5H). A color code reflecting the bioclimatic suitability values at those specific locations was used to show how favorable such sites were for *G. bermejoi*. All the cradles with suitability values below the minimum were removed because *G. bermejoi* populations could not endure such bioclimatic conditions. Our goal here was to assess which cradles could have harbored *G. bermejoi* populations up to the present. The evolution of the suitability values for *G. bermejoi* in the potential LGM and MH cradles was also assessed by means of boxplots (Figure 5A and 5E). It is important to note that on this occasion we used the suitability values for *G. bermejoi* whilst we used those of its parental taxa to locate the potential cradles. Finally, we combined the surviving LGM and MH cradles into a single map. The locations with favorable soils are indicated by the occurrence of the *O. tridentata* [**63-64**] gypsum indicator species (Figure 5I). Due to the dramatic climatic changes in the Iberian Peninsula over the last 25,000 years it is possible that the bioclimatic suitability for *G. bermejoi* may drop abruptly in some potential cradles, areas in which the *G. bermejoi* populations will be doomed. Using these maps it is possible to find out in which potential cradles the conditions might maintain thriving *G. bermejoi* populations to date (Figure 5I). We repeated the process for the MH cradles and in doing so, we can predict the current distribution of *G. bermejoi* based on an assumption of no dispersal, a prediction that can be compared with the actual distribution of this species.

Figure 5. Fate of the potential *G. bermejoi* cradles and expected current distribution of this plant according to our hypothesis. The quaternary climatic oscillations over the past 25,000 years have produced dramatic changes in bioclimatic suitability for this species. In "*A*", the box plots show the bioclimatic suitability values of *G. bermejoi* at the potential LGM cradles at three points in time: the LGM, the MH period and under current climatic conditions. The suitability is almost 0 during the LGM, yet it increases dramatically during the MH period to again drop under current climatic conditions. "*B*" shows all the potential LGM cradles with a color code indicating the bioclimatic suitability values for *G. bermejoi* at such locations (in this case almost 0 at all sites). "*C*" and "*D*" only show the LGM cradles where *G. bermejoi* populations are expected to survive during the MH (*C*) and current (*D*) climatic conditions. We repeated this analysis for the potential cradles produced during the MH period and the results are shown in "E" to "H". This approach enabled us to produce a predicted current distribution for *G. bermejoi*, assuming no dispersal from the original cradles. This expected current distribution is shown in "*I*", along with that of the *O. tridentata* (yellow circles) as an indicator of gypsum soils. Finally, in "*J*" this expected distribution is compared to the current distribution of *G. bermejoi* (red triangles).

Results

MaxEnt models validation

The AUC values obtained in the MaxEnt models were 0.970 (*G. bermejoi*), 0.939 (*G. tomentosa*), 0.905(*G. struthium struthium*) and 0.931 (*G. struthium hispanica*), all sufficiently high to be able to accept such models. The TSS values obtained were 0.810 (*G. bermejoi*), 0.668 (*G. tomentosa*) and 0.584 (*G. struthium struthium*), with TSS values above 0.6 considered good and 0.2-0.6 to be fair-to-moderate.

Location and fate of potential LGM and Mid-Holocene cradles for G. *bermejoi*

We had adopted a two stage strategy to predict the current distribution of G. *bermejoi* based on our hypothesis. First, we located the potential cradles according to the distributions of both parental taxa (*G. struthium struthium* and *G. tomentosa*) during the LGM and the MH periods, and we then assessed in which of those possible cradles the climatic conditions remained reasonably well suited to *G. bermejoi* survival. We obtained several possible centers of formation during the LGM, located in the central region of the Iberian Peninsula, in the Ebro Valley (with some in the north eastern limits of this valley), on the east coast and in the south eastern central region of the Iberian Peninsula (Figures 3C and 5B). However, the bioclimatic suitability for *G. bermejoi* is almost 0 at all of these sites during the LGM (Figures 5A and 5B).

Previous results show that this species has a very narrow bioclimatic niche, preventing its survival during the LGM [**32**]. Thus, it is probable that while the parental taxa exchanged pollen, the climatic conditions repressed speciation [**31**]. This situation changed dramatically following the climate change at the end of the LGM, the MH representing a warmer and more humid period during which the conditions at some of the cradles identified were favorable for *G. bermejoi* populations to thrive (Figure 5C). As such, we found locations with high bioclimatic suitability in the central and eastern regions of the peninsula, and in the middle of the Ebro Valley. In the potential cradles with low suitability values, the climatic conditions make it difficult for such populations to persist. Accordingly, those with values below the survival threshold for *G. bermejoi* were removed.

In the suitability maps for the current climatic conditions, we see that the suitability values dropped abruptly at all the sites around the Ebro Valley, although they remained sufficiently high for *G. bermejoi* to survive at some locations in the Central and Eastern regions of the Peninsula. Those areas are the locations where we would currently expect to find surviving populations of this plant according to the working hypothesis for the LGM cradles. Our map also predicted some locations for *G. bermejoi* in the Ebro Valley, all with suitability values below 0.25. However, if we consider the histograms for *G. bermejoi* suitability (Figures 4A to 4B), we can see that the probability of finding this species at such sites is very low.

The dramatic change in the potential distribution provoked by the climatic transition to the MH period favored different potential cradles for *G. bermejoi*. In this case, the potential cradles were more or less clustered at five locations: the Central, Eastern and South Eastern Region of the Iberian Peninsula; the Ebro Valley; and interestingly, a site in the North West of the peninsula, beyond the Central Mountain System that represents an important geographical barrier. Most of South Eastern areas were ill-fated in terms of *G. bermejoi* survival (Figure 5F) and hence, those below the survival threshold were not considered further (Figure 5G). During the MH period, we saw high bioclimatic suitability at several locations in the Ebro Valley and in the Central, Eastern and North western regions. After this period of benign climatic conditions for *G. bermejoi* at many sites (Figures 5G and 5H), its suitability decreased at many of these locations under current climatic conditions. At present, only some of the cradles in the central and north western sites maintain suitability values that would be compatible with the survival of *G. bermejoi* populations.

The surviving cradles from both the LGM and MH period can be considered (Figure 5I), along with the sites with gypsophyte suitable soils. Most of these gypsum outcrops remain unoccupied by *G. bermejoi* under current climatic conditions, although the models show that this was probably not the case in the past (Figures 5C, 5F and 5G). The surviving cradles can be interpreted as the expected distribution of *G. bermejoi* under conditions where there is absolutely no dispersion (Figure 5I). The occurrence of *G. bermejoi* is shown along with this expected distribution in Figure 5J for comparison (A more detailed map can be seen at the supplementary materials section S2).

Discussion

The results presented here suggest that the current distribution of *G. bermejoi* could be explained, at least in some degree, by our hypothesis. Accordingly, we outline some potential key events concerning the recent evolutionary history of this taxon. In general terms, the bioclimatic suitability for *G. bermejoi* evolved considerably over the last 25,000 years. From the almost null bioclimatic suitability in the LGM, there was a spectacular improvement during the MH period that subsequently decreased as the current climatic conditions were established. Our results show how this trend could have affected the possible formation of *G. bermejoi*, which would not have been present during the LGM. By contrast, several thriving populations of this species could have been established during the benign MH period, thereafter declining as the current climatic conditions set in established. Both parental species follow an almost opposite trend, as during the LGM both *G. struthium* subsp. *struthium* and *G. tomentosa* seem to have been well established in the eastern coastal strip of the Iberian Peninsula. Their ranges have a high degree of sympatry, not least because their bioclimatic niches do not differ [**32**]. Thus, it seems reasonable to us that cross pollination would occur frequently between both the parental taxa, as happens today. However, the climatic conditions in the LGM were not compatible with the survival of the hybrid *G. bermejoi* due to its very narrow bioclimatic range of tolerance [**32**], these conditions repressing its speciation during this period [**31**].

If we consider the LSAs of the parental taxa during the LGM, we see that they overlap at a number of sites with suitable soils. According to our hypothesis, those areas would harbor higher frequencies of autopolyploids of both taxa and they would therefore be potential cradles for this species. While we cannot rule out the formation of *G. bermejoi* in places other than these, the formation rate of hybrid individuals would be lower, as would the probability that stable populations could thrive there. It seems reasonable to accept that the climatic transition to the MH period triggered the formation of the first *G. bermejoi* populations at such sites. We believe that hybrid populations would have blossomed at such locations as soon as the LGM climate changed.

During the MH period, new climatic conditions generated other potential areas for the parental taxa by producing different LSAs for *G. struthium* subsp. *struthium* and *G. tomentosa* and hence, new potential cradles for *G. bermejoi*. We think that the further climatic changes filtered those formation centers and our results indicate that only some of them maintained the bioclimatic conditions for *G. bermejoi* populations to survive. Such surviving populations are located in the Ebro Valley, the central, eastern and south eastern sectors, and interestingly, in the North West beyond the Central mountain range. All the observed sites of *G. bermejoi* are not too distant from the former ones and we think that this distribution is consistent with our hypothesis.

The presence of *G. bermejoi* populations in North west of Spain, beyond the Central System seems difficult to explain in terms of migration. This mountain range is a geographic barrier extending in ENE-WSW direction, with summits reaching heights of 2500 m [**65**] and constituting the boundary between two large tertiary basins: the Duero Basin to the North and the Tagus Basin to the South.

This mountain range was the outcome of deformation, and it developed through triaxial compression that occurred during the Oligocene and the Lower Miocene period (circa 25 million years ago) [**65-66**]. Our hypothesis points to an in situ origin of these populations, because this area matches one of the MH cradles that maintained some degree of climatic suitability up to the present. Other populations near El Rincón de Ademuz are attributed to a more recent introduction [**41**], although a potential cradle is predicted to have survived less than 20 km away from that site. Thus, the presence of *G. bermejoi* there could be understood in the framework of our hypothesis.

Our predictions include some locations in the Ebro valley, although we feel that their low suitability values (bioclimatic suitability < 0.25) might rule out these sites as potential cradles. The histograms showing the bioclimatic suitability at the sites of *G. bermejoi* occurrence according to the MaxEnt models suggest an extremely low frequency of plants below 0.25. Indeed, the species is not currently found in the central Ebro Valley. Moreover, *G. bermejoi* is currently quite dispersed across the Southern Plateau, a flat and windy region. Significantly, the anatomy and structure of *Gypsophila* seeds is appropriate for mid-range dispersal, far from the potential cradles, and these seeds can be released from their capsules by strong winds and animals [**67**]. Indeed, some invasive species of this genus (*G. paniculata*) apparently use a dispersal mechanism based on tumbleweed [**68, 69**].

To conclude, we believe that in conjunction with wind dispersal or other mechanisms, our hypothesis could to some extent explain the current distribution of *G. bermejoi*. However, many questions still remain unanswered regarding the recent evolutionary history of *G. bermejoi*. For instance, the connection between the gypsum outcrops and the dispersion capacity of *G. bermejoi* is unclear, and these data will be crucial to disentangle the recent evolutionary history of this taxon. However, we think that our approach sheds some light on this issue and we encourage research into the genetic structure of the *G. bermejoi* populations, as well as cytogenetic studies to assess the rate and frequency of autopolyploid formation in current LSAs of *G. struthium* subsp. *struthium* and *G. tomentosa*.

Conclusions

According to the results obtained here, we conclude that:

a) The presence of *G. bermejoi* in the Iberian Peninsula can be ruled out during the LGM. Its speciation seems to have been repressed until the end of this climatic period and we have reason to believe that the first populations of this plant started to thrive soon after the end of the LGM (circa 17,000-16,000 years BP).

b) The existence of populations of plants similar to those of the current *G. bermejoi* during the Last Interglacial period cannot be ruled out, although this question is beyond the scope of our study.

c) The overlap between the LSAs for the parental *G. bermejoi* taxa during the LGM may have favored the formation of stable populations of this fertile hybrid soon after the end of this period.

The gypsum outcrops in these areas could represent potential cradles or centers of formation for *G. bermejoi*.

d) Only some of these potential cradles have retained suitable climatic conditions for this plant to date. These cradles that have survived to date may predict the expected distribution of *G. bermejoi*, assuming no dispersion. Such predictions seem to match the true distribution of *G. bermejoi*, particularly if we consider a degree of dispersion by wind or other means. This could explain the presence of this species in the central region of the Iberian Peninsula.

e) Due to the bioclimatic shift, similar potential cradles may have formed in different regions of the Iberian Peninsula during the MH period, which could explain the presence of *G. bermejoi* populations to the North West of the Central mountain range. The presence of these populations is difficult to explain be means of migration, even though it is predicted by our model. If this is true, the north western populations of *G. bermejoi* may be more recent than those located in the central region of the Iberian Peninsula.

f) The hypothesis of higher rates of autopolyploid formation in LSAs could to some extent explain the current distribution of *G. bermejoi*. Although further research will be required to clarify this issue, cytogenetic studies could address this hypothesis, for example assessing the autopolyploid frequencies of *G. struthium* subsp. *struthium* and *G. tomentosa* at their current LSAs.

REFERENCES

1. Adams KL, Wendel JF. Polyploidy and genome evolution in plants. *Current opinion in plant biology*, 2005, vol. 8, no 2, p. 135-141.

2. Otto SP, Whitton J. Polyploid incidence and evolution. *Annu Rev Genet* 2000;34:401-37.

3. Soltis PS, Soltis DE. Ancient WGD events as drivers of key innovations in angiosperms. *Current opinion in plant biology*, 2016, vol. 30, p. 159-165.

4. Tank DC, Eastman JM, Pennell MW, Soltis PS, Soltis DE. Progressive radiations and the pulse of angiosperm diversification. *N Phytol* 2015.

5. Jaillon O et al. The grapevine genome sequence suggests ancestral hexaploidization in major angiosperm phyla. *Nature*, 2007, vol. 449, no 7161, p. 463.

6. Van de Peer Y, Fawcett JA, Proost S, Sterck L & Vandepoele K. The flowering world: a tale of duplications. *Trends in plant science*, 2009, vol. 14, no 12, p. 680-688.

7. Soltis PS, Soltis DE. The role of hybridization in plant speciation. *Annual review of plant biology*, 2009, vol. 60, p. 561-588.

8. Parisod C, Holderegger R, Brochmann C. 2010. Evolutionary consequences of autopolyploidy. *New Phytologist* 186, 5–17.

9. Löve A & Löve D. The significance of differences in the distribution of diploids and polyploids. *Hereditas*, 1943, vol. 29, no 1-2, p. 145-163.

10. Mable BK. Breaking down taxonomic barriers in polyploidy research. *Trends in plant Science*, 2003, vol. 8, no 12, p. 582-590.

11. Grant V. *Plant speciation*. Columbia University Press, 1981.

12. Stebbins GL. Polyploidy, hybridization, and the invasion of new habitats. *Annals of the Missouri Botanical Garden*, 1985, p. 824-832.

13. Brochmann C et al. Polyploidy in arctic plants. *Biological journal of the Linnean society*, 2004, vol. 82, no 4, p. 521-536.

14. Parisod C, Holderegger R & Brochmann C. Evolutionary consequences of autopolyploidy. *New phytologist*, 2010, vol. 186, no 1, p. 5-17.

15. Mable BK. Breaking down taxonomic barriers in polyploidy research. *Trends in plant Science*, 2003, vol. 8, no 12, p. 582-590.

16. Petit C and Thompson J.D. Variation in phenotypic response to light availability between diploid and tetraploid populations of the perennial grass Arrhenatherum elatius from open and woodland sites. *Journal of Ecology*, 1997, p. 657-667.

17. Li WL, Berlyn GP & Ashton PMS. Polyploids and their structural and physiological characteristics relative to water deficit in Betula papyrifera (Betulaceae). *American Journal of Botany*, 1996, vol. 83, no 1, p. 15-20.

18. Wullschleger SD, Sanderson MA, McLaughlin SB, Biradar DP & Rayburn AL. Photosynthetic rates and ploidy levels among populations of switchgrass. *Crop Science*, 1996, vol. 36, no 2, p. 306-312.

19. Del Pozo JC, Ramírez-Parra E. Whole genome duplications in plants: an overview from Arabidopsis. *Journal of Experimental Botany*, 2015, vol. 66, no 22, p. 6991-7003.

20. Fawcett JA, Van de Peer Y. Angiosperm polyploids and their road to evolutionary success. *Trends in Evolutionary Biology*, 2010, vol. 2, no 1, p. 16-21.

21. Ramsey J, Schemske DW. Pathways, mechanisms, and rates of polyploid formation in flowering plants. *Ann Rev Ecol Syst* 1998; 29:467-501.

22. Mason AS, Pires JC. Unreduced gametes: meiotic mishap or evolutionary mechanism?. *Trends in Genetics*, 2015, vol. 31, no 1, p. 5-10.

23. Thompson JD, Lumaret R. The evolutionary dynamics of polyploid plants: origins, establishment, and persistence. *Trends Eco Evol* 1992;7:302-7.

24. Husband BC. Constraints on polyploid evolution: a test of the minority cytotype exclusion principle. *Proceedings of the Royal Society of London. Series B: Biological Sciences* 2000;267:217-23.

25. Barringer BC. Polyploidy and self-fertiliza-tion in flowering plants. *Am J Bot* 2007;94:1527-33.

26. Glennon KL, Ritchie ME & Segraves KA. Evidence for shared broad-scale climatic niches of diploid and polyploid plants. *Ecology letters*, 2014, vol. 17, no 5, p. 574-582.

27. Levin DA. Minority cytotype exclusion in local plant populations. *Taxon*, 1975, p. 35-43.

28. Fowler NL, Levin DA. Ecological constraints on the establishment of a novel polyploid in competition with its diploid progenitor. *The American Naturalist*, 1984, vol. 124, no 5, p. 703-711.

29. Felber F. Establishment of a tetraploid cytotype in a diploid population: effect of relative fitness of the cytotypes. *Journal of Evolutionary Biology*, 1991, vol. 4, no 2, p. 195-207.

30. López G. *Gypsophila bermejoi* G. López, sp. nov. y algunos comentarios sobre el geénero Gypsophila con relación a Flora Iberica. *An Jard Bot Madr*.1984; 41(1): 35–38.

31. de Luis M, Bartolomé C, García-Cardo Ó & Álvarez-Jiménez J. Gypsophila bermejoi G. López: A possible case of speciation repressed by bioclimatic factors. *PloS one*, 2018, vol. 13, no 1, p. e0190536.

32. de Luis M, Bartolomé C, García-Cardo Ó, Martínez-Labarga JM, & Álvarez-Jiménez, J. Sympatric and allopatric niche shift of endemic Gypsophila (Caryophyllaceae) taxa in the Iberian Peninsula. *PloS one*, 2018, vol. 13, no 11, p. e0206043.

33. Smith A, Alsdurf J , Knapp M , Baer SG , Johnson LC . Phenotypic distribution models corroborate species distribution models: A shift in the role and prevalence of a dominant prairie grass in response to climate change . *Global Change Biology* , 2017 . doi: 10.1111/gcb.13666 28211151.

34. Horvitz N, Wang R, Wan FH, Nathan R. Pervasive human-mediated large-scale invasion: analysis of spread patterns and their underlying mechanisms in 17 of China's worst invasive plants . *Journal of Ecology* , 2017 , vol. 105 , no 1 , p. 85 – 94.

35. Bueno ML , Pennington RT , Dexter KG , Kamino LHY , Pontara V , Neves DM , et al . Effects of Quaternary climatic fluctuations on the distribution of Neotropical savanna tree species . *Ecography* , 2017 , vol. 40 , no 3 , p. 403 – 414 .

36. Smeraldo S , Di Febbraro M , Ćirović D , Bosso L , Trbojević I , & Russo D . Species distribution models as a tool to predict range expansion after reintroduction: A case study on Eurasian beavers (Castor fiber) . *Journal for Nature Conservation* , 2017.

37. Zutta BR, Rundel PW. Modeled Shifts in Polylepis Species Ranges in the Andes from the Last Glacial Maximum to the Present . *Forests*, 2017 , vol. 8 , no 7 , p. 232 . doi: 10.3390/f8070232.

38. Bosso L, Luchi N, Maresi G, Cristinzio G, Smeraldo S, Russo D . Predicting current and future disease outbreaks of Diplodia sapinea shoot blight in Italy: species distribution models as a tool for forest management planning . *Forest Ecology and Management*, 2017 , vol. 400 , p. 655 – 664 .

39. Kaki E, Gilbert F. Using species distribution models to assess the importance of Egypt's protected areas for the conservation of medicinal plants . *Journal of Arid Environments* , 2016 , vol. 135 , p. 140 – 146.

40. Rovzar C, Gillespie TW, Kawelo K. Landscape to site variations in species distribution models for endangered plants . *Forest Ecology and Management* , 2016 , vol. 369 , p. 20 – 28 .

41. Mota JF, Sánchez-Gómez P, Guirado JS. Diversidad vegetal de las yeseras ibéricas. El reto de los archipiélagos edáficos para la biología de la conservación. *ADIF-Mediterráneo Asesores Consultores*, Almería, 2011.

42. Mota Poveda JF, Garrido Becerra JA, Pérez-García FJ, Salmerón-Sánchez E, Sánchez-Gómez P, Merlo E. Conceptual baseline for a global checklist of gypsophytes. *Lazaroa*. 2016; 37: 7-30. doi: 10.5209/LAZA.54044

43. Escudero A, et al. Plant life on gypsum: a review of its multiple facets. *Biological Reviews*, 2015, vol. 90, no 1, p. 1-18.

44. Acebes B, Bernabé M., Diaz-Lanza AM, Bartolomé C. Two new sulfated saponins from the roots of Gypsophila bermejoi. *J Nat Prod*. 1998; 61(12): 1557-1559. doi: 10.1021/np9705221

45. Acebes B, Daz-Lanza AM, Bernabé M. A saponin from the roots of Gypsophila bermejoi. *Phytochemistry*. 1998; 49(7): 2077-2079. doi: 10.1016/S0031-9422(98)00404-X

46. Boscaiu M, Sánchez M, Bautista I, Donat P, Lidón A, Llinares J, Bartolomé C, Vicente O. Phenolic compounds as stress markers in plants from gypsum habitats. Bull Univ Agric Sci Vet Med Cluj Napoca, *Horticulture*. 2010; 67(1): 1843-5254. doi: 10.15835/buasvmcn-hort:4903

47. *G.tomentosa*: GBIF.org (10th November 2016) GBIF Occurrence Download http://doi.10.15468/dl.my22ws. *G. struthium subs. struthium*:GBIF.org (11th November 2016) GBIF Occurrence Download http://doi.10.15468/dl.dzjhbc. *G. bermejoi*:GBIF.org (11th November 2016) GBIF Occurrence Download http://doi.10.15468/dl.engt9m

48. Hijmans RJ et al. Very high resolution interpolated climate surfaces for global land areas. International *Journal of Climatology*, 2005, vol. 25, no 15, p. 1965-1978.

49. Gent PR. The community climate system model version 4. *Journal of Climate*, 2011, vol. 24, no 19, p. 4973-4991.

50. Merow C, Smith MJ, Silander JA. A practical guide to MaxEnt for modeling species' distributions: what it does, and why inputs and settings matter. *Ecography*, 2013, vol. 36, no 10, p. 1058–1069.

51. Pueyo Y & Alados CL. Abiotic factors determining vegetation patterns in a semi-arid Mediterranean landscape: different responses on gypsum and non-gypsum substrates. *Journal of Arid Environments*, 2007, vol. 69, no 3, p. 490-505.

52. Dodd MB & Lauenroth WK. The influence of soil texture on the soil water dynamics and vegetation structure of a shortgrass steppe ecosystem. *Plant Ecology*, 1997, vol. 133, no 1, p. 13-28.

53. Phillips SJ, Anderson RP, Schapire RE. Maximum entropy modeling of species geographic distributions. *Ecological modelling*, 2006, vol. 190, no 3, p. 231-259.

54. Phillips SJ, Dudík M. Modeling of species distributions with Maxent: new extensions and a comprehensive evaluation. Ecography, 2008, vol. 31, no 2, p. 161-175.

55. R Core Team (2016). R: A language and environment for statistical computing. R Foundation for Statistical Computing, Vienna, Austria. URL https://www.R-project.org/.

56. Elith J et al. A statistical explanation of MaxEnt for ecologists. *Diversity and distributions*, 2011, vol. 17, no 1, p. 43-57.

57. Merow C, Smith MJ, Silander JA. A practical guide to MaxEnt for modeling species' distributions: what it does, and why inputs and settings matter. *Ecography*, 2013, vol. 36, no 10, p. 1058-1069.

58. Allouche O, Tsoar A, Kadmon R. Assessing the accuracy of species distribution models: prevalence, kappa and the true skill statistic (TSS). *Journal of Applied Ecology*, 2006, vol. 43, no 6, p. 1223–1232.

59. Fielding AH, Bell JF. A review of methods for the assessment of prediction errors in conservation presence/absence models. *Environmental Conservation*, 1997, vol. 24, no 1, p. 38.

60. Husband, B. C. (2004). The role of triploid hybrids in the evolutionary dynamics of mixed-ploidy populations. *Biological Journal of the Linnean Society*, *82*(4), 537-546.

61. Felber F & Bever JD. (1997). Effect of triploid fitness on the coexistence of diploids and tetraploids. *Biological Journal of the Linnean Society*, *60*(1), 95-106.

62. QGIS Development Team, 2009. QGIS Geographic Information System. Open Source Geospatial Foundation. URL http://qgis.osgeo.org.

63. Ferrandis P, Herranz J M, Copete MA. Caracterización florística y edáfica de las estepas yesosas de Castilla-La Mancha. *Investigación Agraria: Sistemas y Recursos Forestales,* 2005, vol. 14, p. 195-216.

64. Martínez-Hernández F et al. Preliminary essay on the chorology of the Iberian gypsicolous flora: rarity and richness of the gypsum outcrops. *Acta Botanica Gallica*, 2009, vol. 156, no 1, p. 9-18.

65. De Vicente G, et al. Cenozoic thick-skinned deformation and topography evolution of the Spanish Central System. *Global and Planetary Change*, 2007, vol. 58, no 1-4, p. 335-381.

66. Vergés J, Fernández M. Ranges and basins in the Iberian Peninsula: their contribution to the present topography. *Geological Society, London, Memoirs*, 2006, vol. 32, no 1, p. 223-234.

67. García Casanova M. Germinación de semillas, desarrollo de plántulas y niveles de prolina en dos especies del género Gypsophila. 2011. Tesis Doctoral.

68. Leimbach-Maus HB, Parks SR, Partridge CG. Genetic structure of invasive babys breath (Gypsophila paniculata) populations in a freshwater Michigan dune system. *bioRxiv*, 2018, p. 401950.

69. Darwent AL, Coupland R.T. Life history of Gypsophila paniculata. *Weeds*, 1966, vol. 14, no 4, p. 313-318.]

Figure 1

Click here to download Figure Fig_1.tif

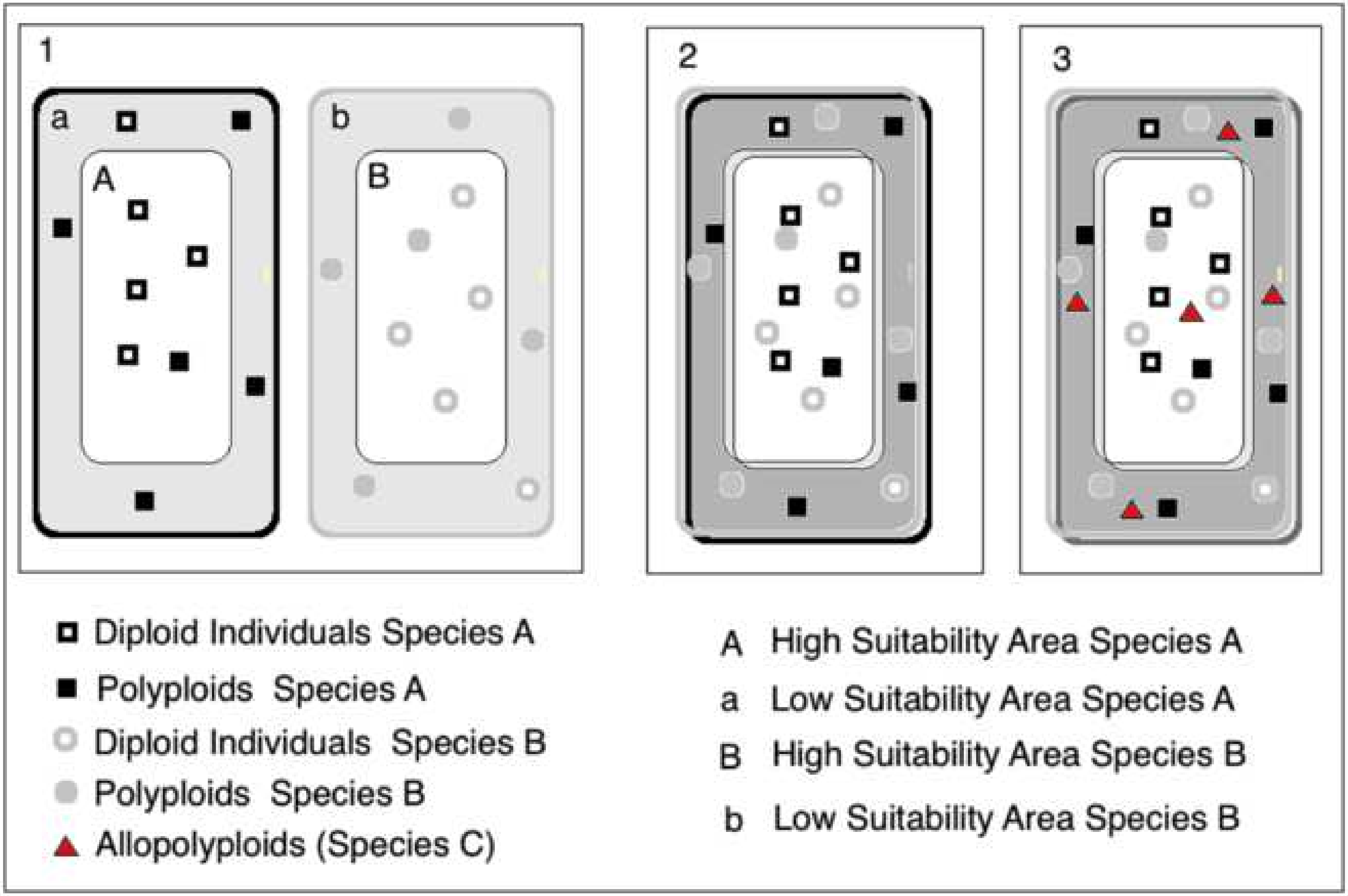

Figure 2

Click here to download Figure Fig_2.tif

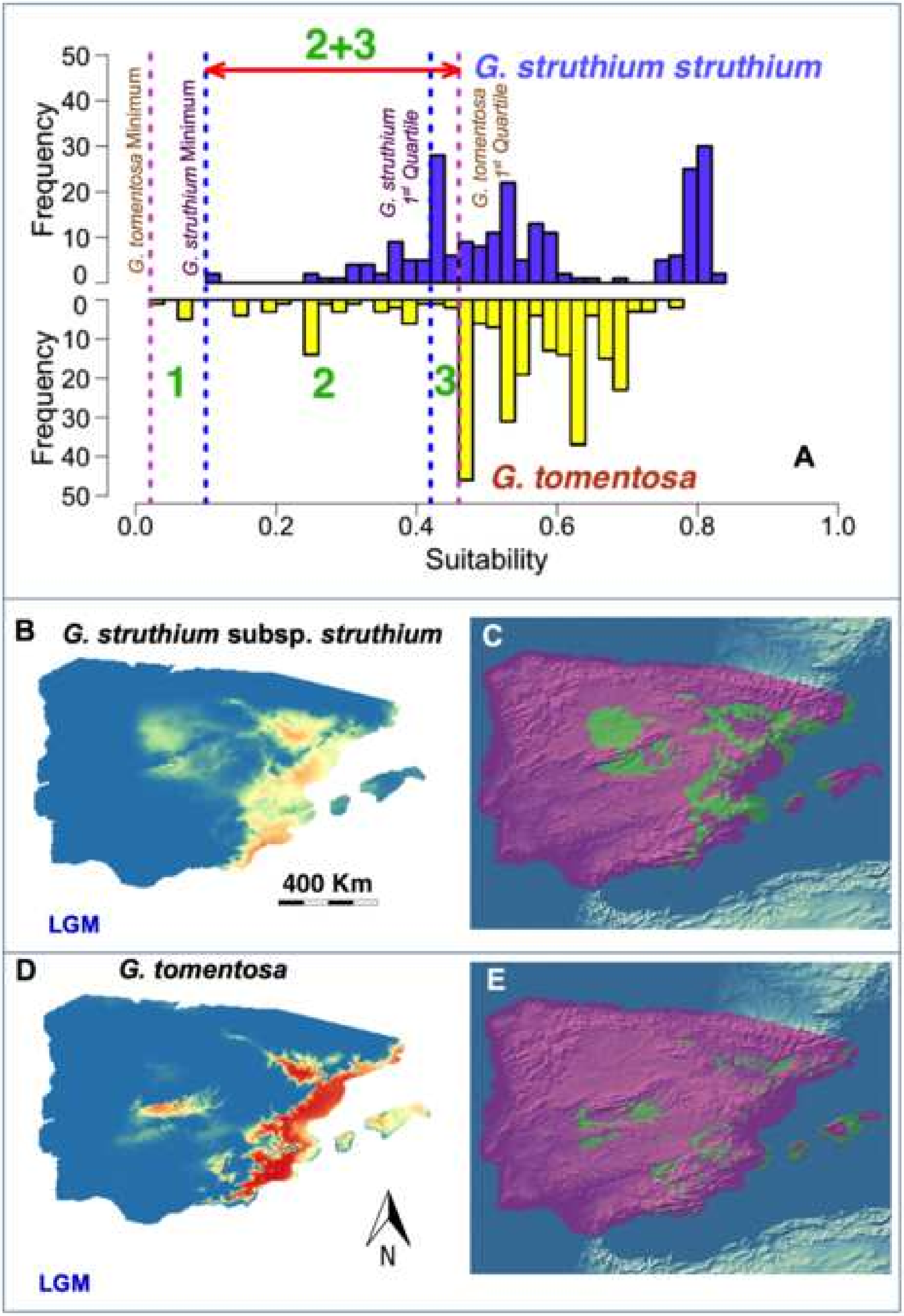

Figure 3

Click here to download Figure Fig_3.tif

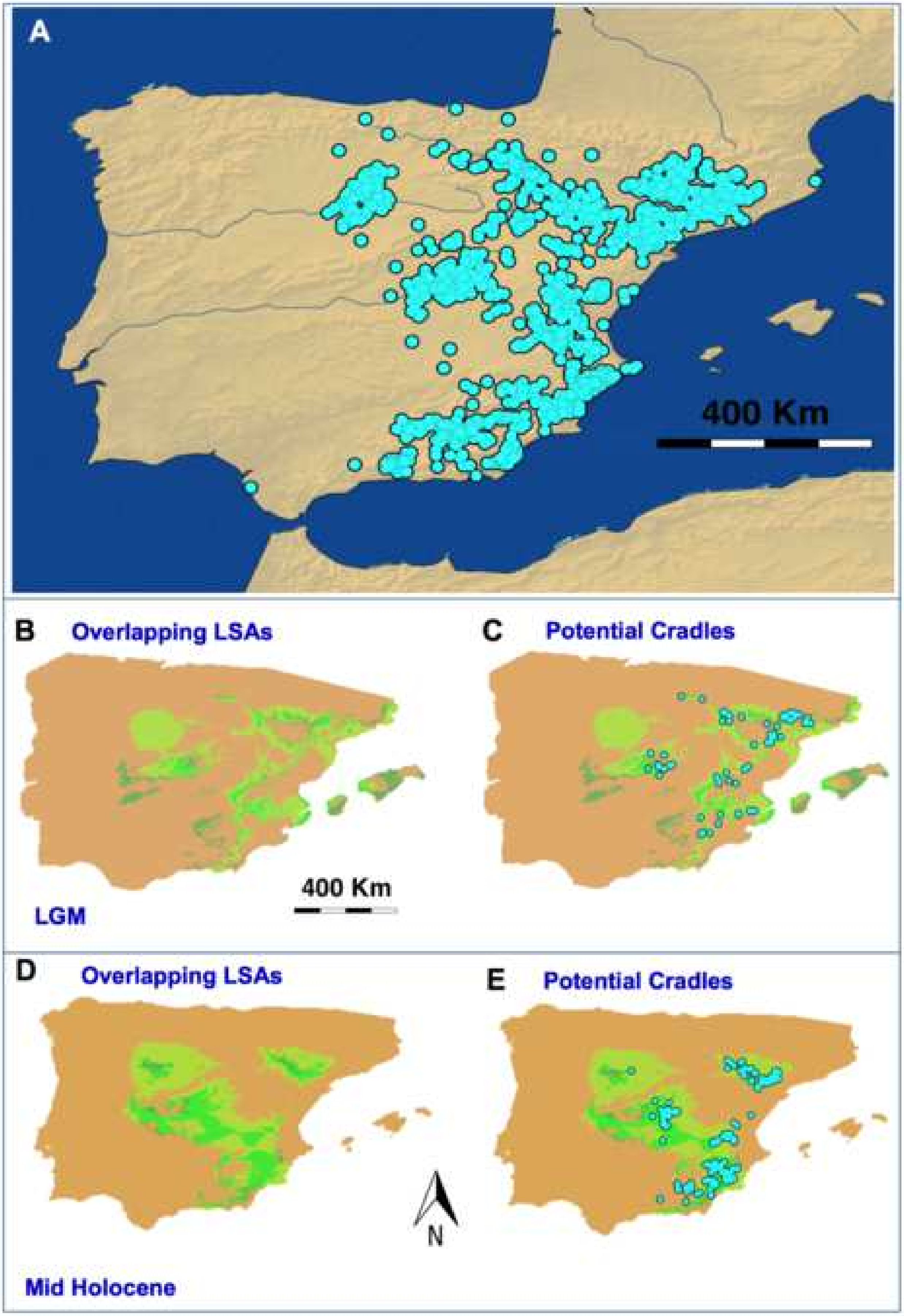

Figure 4

Click here to download Figure Fig_4.tif

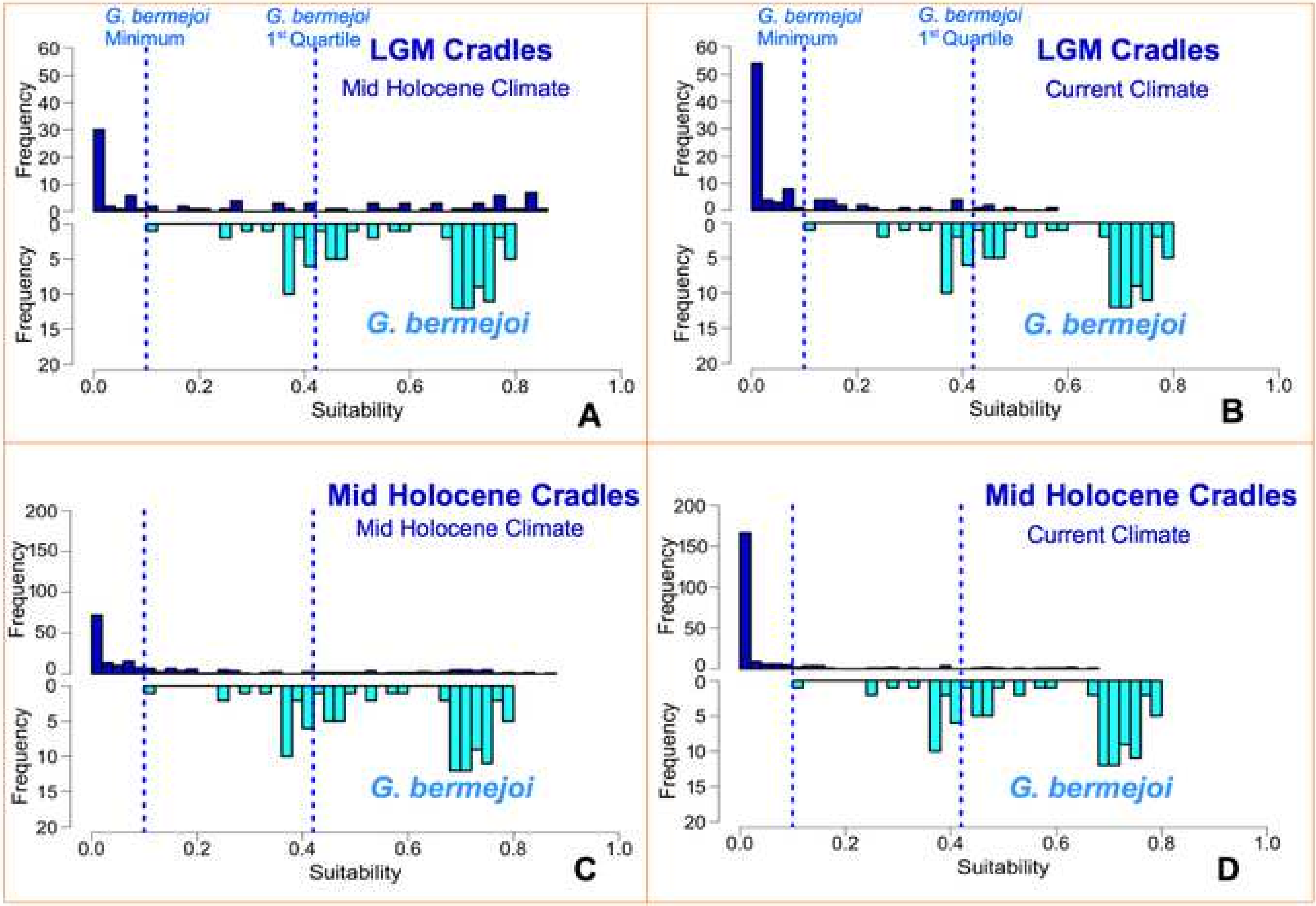

Figure 5

Click here to download Figure Fig_5.tif

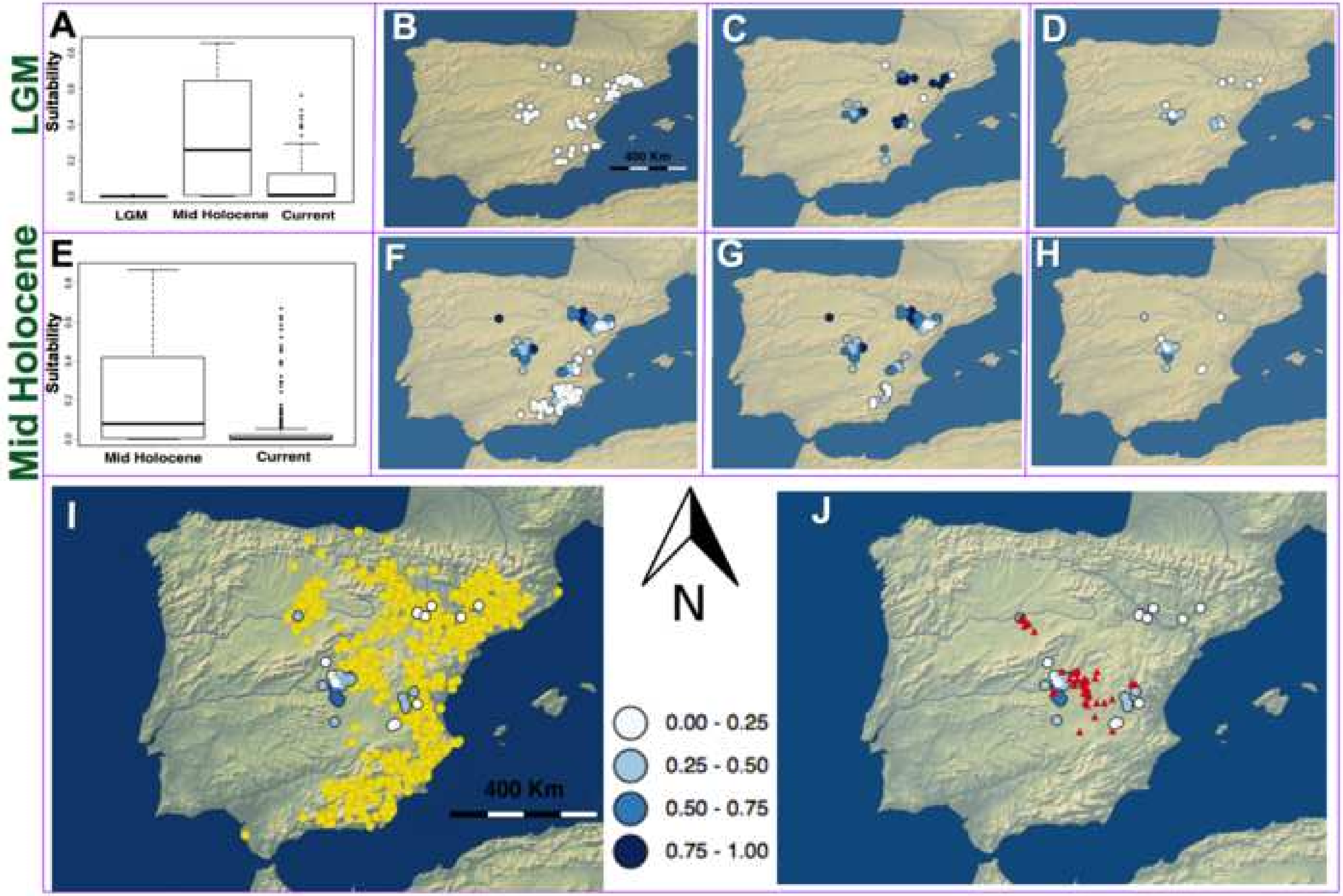

Supplementary material

Click here to access/download
Supporting Information
S1.tif
