# Supplementary material for: Using species distribution models to locate the potential cradles of the allopolyploid Gypsophila bermejoi G. López (Caryophyllaceae)
Source: PLoS One. 2020 May 19;15(5):e0232736. doi: 10.1371/journal.pone.0232736 (PMC7237017; doi:10.1371/journal.pone.0232736)
Supplement: Attachment — Submitted filename: Response to Reviewers.docx [file pone.0232736.s005.docx]

Dear all, editors and reviewers

The following letter gives an answer to all the changes and explanations requested.

Response to the requirements of the journal and reviewers:

1. We have done again all the models, calculations and maps from scratch. We performed ensembles of models generated using four different algorithms (General Linear Models, MaxEnt, Random Forest and MARS) with 30 bootstrap replications each.
2. Ensembles using Three General Circulation Models were performed for the projections to the Mid Holocene and Last Glacial Maximum for each species.
3. The Uncertainties were assessed and mapped at the former steps.
4. Gypsum soils maps were used in spite of using the indicator species *Ononis tridentata*.
5. Several survival tresholds for *Gypsophila bermejo*i were explored to assess their effects on the results.
6. We have revised all the references suggested and taken them into account to improve our research.
7. All the small comments have been addressed.

**to Reviewers 1 and 2**

We are grateful for the comments made on our study and we appreciate sincerely the encouraging comments. We hope you find all the topics complete and clear with all the information added in this revised manuscript.

We appreciate your feedback and hope to have responded effectively.

Kind regards

The authors
