# Supplementary material for: Using species distribution models to locate the potential cradles of the allopolyploid Gypsophila bermejoi G. López (Caryophyllaceae)
Source: PLoS One. 2020 May 19;15(5):e0232736. doi: 10.1371/journal.pone.0232736 (PMC7237017; doi:10.1371/journal.pone.0232736)
Supplement: Attachment — Submitted filename: Response to Reviewers2.docx [file pone.0232736.s006.docx]

Dear all, editors and reviewers

The following letter gives an answer to all the changes and explanations requested.

Response to the requirements of the journal and reviewers:

1. We have corrected all the miswritten words.
2. Sentences at lines 216, 233 and 359 have been corrected. Those lines mention MaxEnt as the only algorithm used and are remnants of the first version of the manuscript. For the current version all the calculations were repeated from scratch using four algorithms, and three General Circulation Models for the projections to the LGM and Mid Holocene.

**to Reviewer 1**

Again, we are grateful for your encouraging comments. All the small comments have been addressed, including the corrections of the miswritten words and the sentences at lines 216, 233 and 359.

We appreciate your feedback and hope to have responded effectively.

**to Reviewer 3**

We appreciate sincerely the comments made on our study.

All the mistakes have been corrected, including sentences at lines 216, 233 and 359 which state that MaxEnt was the only algorithm used, which is not true in this new version. Those lines were remnants of the previous version of the manuscript.

In the current version we state clearly that four algorithms were used to perform the ensembles and, later, three General Circulation models were used for the projections to the LGM and Mid Holocene.

We hope you find all the topics complete and clear with all the information added in this revised manuscript.

Kind regards

The authors
